# Supplementary material for: Transcriptome Analyses in a Selected Gene Set Indicate Alternative Oxidase (AOX) and Early Enhanced Fermentation as Critical for Salinity Tolerance in Rice
Source: Plants (Basel). 2022 Aug 18;11(16):2145. doi: 10.3390/plants11162145 (PMC9415304; doi:10.3390/plants11162145)
Supplement: Supplementary file 1 [file plants-11-02145-s001.zip › Supplementary Table S1.pdf]

Supplementary Table S1. List of *Oryza sativa* genes used to advance in cell reprograming under salt stress.

Function	Gene	Name in NCBI	Name (Based in phylogeny)	Acession number (mRNA)
Glycolysis	cytosolic PFK (Total PFK)		Os-PFK-2	XM_015755545.2
			Os-PFK-2L	XM_015779933.2
			Os-PFK-3	XM_015786994.2
			Os-PFK-6	XM_015784222.2
			Os-PFK-6a	XM_015766054.2
			Os-PFK-6b	XM_015768974.2
			Os-PFK-6c	XM_015782143.2
Fermentation	Total ADH		Os-ADH-1a	XM_015761367.2
			Os-ADH-1b	XM_015761366.2
			Os-ADH-1c	XM_015761368.2
			Os-ADH-2	XM_015771683.1
ROS formation control	Total AOX		Os-AOX1a	XM_015779927.2
			Os-AOX1c	XM_015768323.2
			Os-AOX1d	XM_015778145.2
			Os-AOX1e	XM_015768313.2
	Total UCP		Os-UCP1	XM_015761308.2
			Os-UCP2	XM_015766715.2
	PTOX		Os-PTOX	XM_015777974.2
ROS scavenger	cytosolic APX	Os-APX-1	Os-APX-1	XM_015775012.2
		Os-APX-2	Os-APX-2	XM_015791070.2
	peroxisomal APX	Os-APX-4	Os-APX-3a	XM_015795322.2
			Os-APX-3b	XM_015756537.2
		Os-APX-3	Os-APX-5	XM_015778949.2
	mitochondrial APX	Os-APX-5	Os-stAPX-a	XM_015762991.2
		Os-APX-6	Os-stAPX-b	XM_015762689.2
	Plastidial APX		Os-APX-4	XM_015780570.1
	Plastid.mito APX	Os-APX-7	Os-stAPX-c	XR_001545414.1
		Os-APX-8	Os-stAPX-d	XM_015767619.2
			Os-APX-6	XM_015795334.2
	cytosolic MDHAR		Os-MDHAR-1	XM_015756280.2
			Os-MDHAR-2	XM_015794451.2
	peroxisomal MDHAR;		Os-MDHAR-1	XM_015756280.2
	plastidial MDHAR		Os-MDHAR-4a	XM_015771553.2
			Os-MDHAR-4b	XM_015771554.2
	plastid.mito MDHAR		Os-MDHAR-6	XM_026020028.1
	cyto.peroxi DHAR		Os-DHAR-1	XM_015783968.1
	plastid.mito DHAR		Os-DHAR-3	XM_015785549.2
	cyto.peroxi GR		Os-GR-1	XM_015771322.1

	plastid.mito GR		Os-GR-2a	XM_015772374.2
			Os-GR-2b (3)	XM_015757646.2
	cytosolic SOD		Os-Cu/Zn-SOD-1a	XM_015777122.2
			Os-Cu/Zn-SOD-1b	XM_015792285.2
			Os-Cu/Zn-SOD3	XM_015775916.2
	peroxisomal SOD		Os-Cu/Zn-SOD3	XM_015775916.2
	plastid SOD		Os-Cu/Zn-SOD2	XM_015794032.2
			Os-Fe-SOD1	XM_015785755.1
			Os-Fe-SOD3	XM_015787009.1
			Os-chaperone-SOD	XM_015780024.2
	mitochondrial SOD		Mn-SOD	XM_015784641.2
	cytosolic GPX		Os-GPX1	XM_015787450.2
			Os-GPX3	XM_015759999.2
	plastidial GPX		Os-GPX4	XM_015777305.2
			Os-GPX6b	XM_015780454.2
	mitochondrial GPX		Os-GPX6a	XM_015767260.1
	Total CAT	CATB	Os-CAT1	XM_015787591.2
		CATC	Os-CAT2	XM_015774263.2
		CATA	Os-CAT3	XM_015769909.2
Cell respiration	Total COX		Os_COX1	XM_015763180.2
			Os_COX-5b1	XM_015787012.2
			Os_COX-5b2	XM_015781477.2
			Os_COX-5c	XM_015764388.2
			Os_COX-6a	XM_015777610.2
			Os_COX-6b1	XM_015775050.2
			Os_COX-6b1.1	XM_015790153.2
			Os_COX-6b2.1	XM_015778339.2
			Os_COX-6b2	XM_015769438.2
			Os_COX-x	XM_015789233.2
			Os_COX-y	XM_015794624.2
