# Supplementary material for: Transcriptome Analyses in a Selected Gene Set Indicate Alternative Oxidase (AOX) and Early Enhanced Fermentation as Critical for Salinity Tolerance in Rice
Source: Plants (Basel). 2022 Aug 18;11(16):2145. doi: 10.3390/plants11162145 (PMC9415304; doi:10.3390/plants11162145)
Supplement: Supplementary file 1 [file plants-11-02145-s001.zip › Supplementary Table S2.pdf]

Supplementary Table S2. Predict /experimental subcellular localization of antioxidant proteins from *Oryza sativa.*

Antioxidant proteins	*Oryza sativa*						
	Predict Subcellular Localization						
	Protein	TargetP 2.0	MitoProtII	DeepLoc-1.0	Plant-mSubP*	Experimental confirmation	Consensus
APX	APX-1	Other (99%)	-	Cyto (73%)	Cyto (60%)	Cyto (Caverzan, 2012)	Cyto
	APX-2	Other (99%)	-	Cyto (64%)	Cyto (58%)	Cyto (Guan et al., 2012; Caverzan, 2012)	Cyto
	APX-3a	Other (97%)	-	Peroxi (86%)	Mito (19%) Peroxi (16%)	Peroxi (Teixeira et al., 2006; Caverzan, 2012))	Peroxi
	APX-3b	Other (98%)	-	Peroxi (87%)	Peroxi (23%) Cyto (9%)		Peroxi
	APX-4	Chlo (99%)	-	Plastid (99%)	Plastid (87%)		Chlo
	APX-5	Other (99%)	-	Peroxi (80%)	Cyto (22%) Peroxi (10%)	Peroxi (Teixeira et al., 2006; Caverzan, 2012)	Peroxi
	APX-6	Chlo (94%)	M (99%)	Plastid (93%)	Plastid (85%) Mito.Plastid (10%)		Plastid/Mito
	stAPXa	Mito (97%)	M (96%)	Mito (61%) Extracellular (14%) Plastid (10%)	Mito (46%)	Plastid/Mito (Caverzan, 2012)	Plastid/Mito
	stAPXb	Mito (96%)	M (99%)	Mito (48%) Extracellular (19%) Plastid (11%)	Mito (42%)	Mito (Teixeira et al., 2006; Caverzan, 2012)	Mito
	stAPXc	Chlo (95%)	M (92%)	Plastid (58%) Mito (41%)	Plastid (85%)	Plastid (Caverzan, 2012)	Plastid/Mito
	stAPXd	Chlo (96%)	M (90%)	Plastid (58%) Mito (42%)	Plastid (92%)		Plastid/Mito
MDHAR	MDHAR-1	Other (99%)	-	Peroxi (92%) Cyto (6%)	Cyto (74%)		Peroxi/Cyto
	MDHAR-2	Other (86%)	-	Peroxi (92%) Cyto (7%)	Cyto (63%)		Peroxi/Cyto
	MDHAR-4a	Other (98%)	-	Peroxi (94%) Mito (5%)	Plastid (44%)	Plastid (Liu et al., 2018)	Plastid
	MDHAR-4b	Other (67%)/	-	Peroxi (89%) Mito (10%)	Plastid (32%)	Plastid (Liu et al., 2018)	Plastid

		Mito (17%)					
	MDHAR-6	Mito (47%)/ Chlo (29%)	M (55%)	Plastid (61%) Mito (27%)	Mito.Plastid (56%)	Plastid/Mito (Morgante et al., 2009)	Plastid/Mito
DHAR	DHAR-1	Other (99%)	-	Cyto (60%) Peroxi (10%)	Cyto (63%)		Peroxi/Cyto
	DHAR-3	Chlo (99%)	M (99%)	Plastid (99%)	Plastid (96%) Mito. Plastid (1.2%)		Plastid/Mito
GR	GR-1	Other (99%)	-	Mito (35%) Peroxi (32%) Cyto (26%)	Cyto (72%) Peroxi (4%)		Peroxi/Cyto
	GR-2a	Chlo (98%)	M (99%)	Plastid (99%)	Plastid (80%) Mito (9%)	Plastid/Mito (Wu et al., 2013)	Plastid/Mito
	GR-2b	Chlo (96%)	M (99%)	Plastid (99%)	Plastid (94%) Mito (2%)	Plastid/Mito (Wu et al., 2013)	Plastid/Mito
SOD	Cu/Zn-SOD1a	Other (68%)	-	Cyto (61%) Peroxi (27%) Mito (9%)	Cyto (79%)		Cyto
	Cu/Zn-SOD1b	Other (58%)	-	Cyto (61%) Peroxi (27%) Mito (10%)	Cyto (82%)		Cyto
	Cu/Zn-SOD2	Chlo (99%)	M 78%	Plastid (99%)	Plastid (92%)	Plastid (Guan et al., 2017)	Plastid
	Cu/Zn-SOD3	Other (86%)	-	Cyto (41%) Peroxi (42%)	Cyto (38%)		Peroxi/Cyto
	Mn-SOD1	Mito (81%)	M (93%)	Mito (99%)	Mito (48%)		Mito
	Chaperone-SOD	Chlo (91%)	M (50%)	Plastid (98%)	Plastid (76%)		Plastid
	Fe-SOD1	Chlo (99%)	M (96%)	Plastid (99%)	Plastid (81%)		Plastid
	Fe-SOD3	Chlo (92%)	M (95%)	Plastid (98%)	Plastid (69%)	Plastid (Wang et al., 2021)	Plastid
CAT	CAT1	Other (99%)	-	Peroxi (53%) cyto (32%) Mito (6%)	Peroxi (51%)	Peroxi (Zhang et al., 2016)	Peroxi
	CAT2	Other (99%)	-	Peroxi (53%) cyto (29%) Mito (6%)	Peroxi (48%)	Peroxi (Zhang et al., 2016)	Peroxi
	CAT3	Other (99%)	-	Peroxi (56%) cyto (29%) Mito (6%)	Golgi (34%) Plastid (20%)	Cyto (Zhang et al., 2016)	Cyto
GPX	GPX1	Chlo (99%)	M (97%)	Plastid (93%)	Plastid (77%)		Plastid
	GPX3	Signal peptide (90%)	M (26%)	Plastid (97%)	Plastid (97%)		Plastid
	GPX4	Other (99%)	-	Cyto (74%)	Cyto (82%)		Cyto

	GPX6a	Mito (48%)/ Chlo (38%)	M (99%)	Plastid (88%) Mito (10%)	Mito.Plastid (68%)		Mito.Plasti d
	GPX6b	Other (99%)	-	Cyto 79%	Cyto (42%)		Cyto

Abbreviations: Cyto: cytoplasm; Peroxi: peroxisome; Mito: mitochondria. *Prediction approach followed: Hybrid PseAAC, NCC and Dipep.
