# Supplementary material for: Transcriptome Analyses in a Selected Gene Set Indicate Alternative Oxidase (AOX) and Early Enhanced Fermentation as Critical for Salinity Tolerance in Rice
Source: Plants (Basel). 2022 Aug 18;11(16):2145. doi: 10.3390/plants11162145 (PMC9415304; doi:10.3390/plants11162145)
Supplement: Supplementary file 1 [file plants-11-02145-s001.zip › Supplementary Table S3.pdf]

Supplementary Table S3. Means of RPKM values ± SD (standard deviation) of antioxidant transcripts during salt stress in rice genotype (Pokkali). Statistical analysis (t test) was applied in relation to the controls (water) of each time point 1, 2, 5, 10 or 24 h. Up and down regulated genes are in green and red, respectively. Significant differences from the controls are indicated by * at $p < 0.05$.

Genotype: Pokkali						
	**1 h**		**2h**		**5 h**	
	**Control**	**Salt**	**Control**	**Salt**	**Control**	**Salt**
Cyto-SOD	645,85 ± 5,58	482,21 ± 33,79*	516,09 ± 19,83	520,21 ± 34,00	699,42 ± 64,64	569,63 ± 10,70*
Peroxi-SOD	72,36 ± 8,12	62,10 ± 5,32	87,54 ± 11,02	86,43 ± 4,94	130,72 ± 11,29	114,88 ± 9,82
Plastid-SOD	381,61 ± 31,42	332,65 ± 19,15	313,10 ± 2,44	278,86 ± 13,85	380,40 ± 11,19	376,16 ± 25,90
Mito-SOD	67,10 ± 12,68	51,25 ± 14,31	76,35 ± 7,66	69,69 ± 6,39	124,32 ± 9,12	110,77 ± 19,34
Cyto-APX	423,40 ± 43,89	392,89 ± 62,17	248,33 ± 48,57	266,27 ± 45,71	338,35 ± 40,73	326,25 ± 31,19
Cyto-MDHAR	239,64 ± 21,77	190,03 ± 19,25	144,32 ± 11,71	149,04 ± 29,75	177,78 ± 13,78	141,07 ± 31,65
Cyto.Peroxi-DHAR	202,29 ± 7,83	257,23 ± 3,03	206,46 ± 8,26	295,37 ± 40,44*	189,78 ± 23,58	241,64 ± 39,08
Cyto.Peroxi-GR	53,18 ± 5,76	48,71 ± 3,76	52,64 ± 4,99	52,30 ± 3,02	51,18 ± 3,15	50,58 ± 1,19
Peroxi-APX	146,35 ± 2,49	147,98 ± 8,34	214,78 ± 17,80	207,68 ± 5,61	171,75 ± 5,29	162,58 ± 12,87
Peroxi-MDHAR	348,50 ± 23,31	315,54 ± 20,03	256,19 ± 17,19	255,15 ± 45,08	248,66 ± 16,14	213,10 ± 37,51
Plastid-APX	66,63 ± 5,31	66,96 ± 7,58	65,66 ± 3,53	59,03 ± 6,37	67,26 ± 3,96	65,40 ± 3,39
Plastid-MDHAR	141,59 ± 5,23	155,34 ± 7,09	145,00 ± 10,47	135,75 ± 15,89	108,56 ± 4,33	104,19 ± 11,16
Mito-APX	15,99 ± 3,73	12,26 ± 0,59	17,87 ± 1,12	15,19 ± 1,31	31,16 ± 0,67	24,74 ± 3,93
Plasti.Mito APX	330,66 ± 35,46	327,73 ± 15,42	425,79 ± 18,40	383,00 ± 54,50	433,76 ± 78,77	394,00 ± 25,05
Plastid.Mito-MDHAR	32,72 ± 3,33	29,83 ± 4,25	33,12 ± 3,97	29,64 ± 2,35	37,68 ± 2,57	32,16 ± 0,91
Plastid.Mito-DHAR	69,48 ± 12,75	66,79 ± 20,69	107,50 ± 27,81	114,31 ± 11,65	117,79 ± 14,07	126,44 ± 27,13
Plastid.Mito-GR	22,96 ± 2,73	18,45 ± 1,07	26,60 ± 1,69	21,81 ± 1,27	27,07 ± 1,42	26,49 ± 2,10
Cyto-GPX	234,14 ± 13,93	210,30 ± 22,14	324,68 ± 18,67	302,36 ± 33,80	226,05 ± 17,14	202,08 ± 19,98
Plastid-GPX	825,84 ± 51,60	648,84 ± 106,72*	981,18 ± 53,56	806,36 ± 50,07*	755,99 ± 42,87	650,41 ± 152,31*
Mito.Plastid-GPX	78,37 ± 19,91	70,95 ± 11,60	72,33 ± 5,83	74,94 ± 17,04	95,52 ± 8,25	80,24 ± 17,32
Cyto-Cat	19,93 ± 6,98	23,37 ± 0,79	23,09 ± 0,37	11,12 ± 2,09	32,76 ± 0,10	60,81 ± 23,86
Peroxi-Cat	292,81 ± 12,66	303,37 ± 32,45	245,61 ± 53,34	211,91 ± 13,49	143,42 ± 26,53	173,66 ± 13,80

Genotype: Pokkali				
	10 h		24 h	
	Control	Salt	Control	Salt
Cyto-SOD	554,06 ± 100,23	563,38 ± 19,24	741,73 ± 56,54	682,05 ± 85,09
Peroxi-SOD	85,10 ± 11,33	93,08 ± 11,21	102,19 ± 5,72	86,45 ± 13,82
Plastid-SOD	463,90 ± 15,88	446,11 ± 5,66	263,07 ± 29,38	308,07 ± 23,84
Mito-SOD	142,08 ± 11,12	152,59 ± 13,01	74,46 ± 13,54	72,84 ± 13,12
Cyto-APX	467,37 ± 34,85	574,56 ± 20,59*	315,81 ± 47,01	439,10 ± 57,67*
Cyto-MDHAR	160,15 ± 8,40	174,18 ± 17,51	173,77 ± 19,23	203,28 ± 11,05
Cyto.Peroxi-DHAR	196,64 ± 12,78	195,52 ± 22,35	138,52 ± 12,18	265,48 ± 34,88*
Cyto.Peroxi-GR	59,32 ± 1,62	64,97 ± 2,84	38,66 ± 3,43	53,42 ± 2,38
Peroxi-APX	189,33 ± 21,09	181,31 ± 18,15	174,44 ± 36,26	201,53 ± 50,85
Peroxi-MDHAR	215,44 ± 13,03	228,45 ± 14,54	247,29 ± 62,77	267,22 ± 22,07
Plastid-APX	53,34 ± 2,68	58,05 ± 1,94	42,27 ± 7,74	52,48 ± 6,75
Plastid-MDHAR	95,91 ± 6,98	87,30 ± 5,22	90,63 ± 47,37	79,34 ± 15,46
Mito-APX	35,59 ± 3,56	35,76 ± 2,64	19,64 ± 3,11	13,55 ± 2,87
Plasti.Mito APX	444,11 ± 32,85	451,22 ± 44,87	339,70 ± 74,13	465,08 ± 1,51*
Plastid.Mito-MDHAR	40,63 ± 2,44	33,02 ± 5,36	17,11 ± 1,76	15,40 ± 1,35
Plastid.Mito-DHAR	102,51 ± 14,66	129,10 ± 25,93	56,87 ± 3,90	67,77 ± 13,64
Plastid.Mito-GR	37,25 ± 2,33	38,60 ± 3,73	12,66 ± 0,18	14,37 ± 2,72
Cyto-GPX	64,37 ± 10,42	94,52 ± 6,10	205,81 ± 50,82	272,48 ± 58,36
Plastid-GPX	542,80 ± 58,59	541,07 ± 45,85	1310,75 ± 208,75	1183,61 ± 112,57*
Mito.Plastid-GPX	85,09 ± 3,37	85,19 ± 8,08	88,00 ± 10,34	117,01 ± 11,03
Cyto-Cat	48,25 ± 9,34	56,93 ± 12,07	11,13 ± 0,44	1,05 ± 0,36
Peroxi-Cat	154,85 ± 17,63	172,54 ± 3,33	71,38 ± 15,86	135,68 ± 21,85*
