# Supplementary material for: Transcriptome Analyses in a Selected Gene Set Indicate Alternative Oxidase (AOX) and Early Enhanced Fermentation as Critical for Salinity Tolerance in Rice
Source: Plants (Basel). 2022 Aug 18;11(16):2145. doi: 10.3390/plants11162145 (PMC9415304; doi:10.3390/plants11162145)
Supplement: Supplementary file 1 [file plants-11-02145-s001.zip › Supplementary Table S4.pdf]

Supplementary Table S4. Means of RPKM values ± SD (standard deviation) of antioxidant transcripts during salt stress in rice genotype (ir29). Statistical analysis (t test) was applied in relation to the controls (water) of each time point 1, 2, 5, 10 or 24 h. Up and down regulated genes are in green and red, respectively. Significant differences from the controls are indicated by * at $p < 0.05$.

Genotype: ir29						
	**1 h**		**2h**		**5 h**	
	**Control**	**Salt**	**Control**	**Salt**	**Control**	**Salt**
Cyto-SOD	610,69 ± 21,79	535,95 ± 32,89	479,61 ± 23,90	596,73 ± 32,10*	511,42 ± 19,25	481,44 ± 26,73
Peroxi-SOD	74,18 ± 6,67	69,82 ± 1,56	58,45 ± 9,21	68,28 ± 3,93	78,30 ± 6,43	79,11 ± 15,29
Plastid-SOD	319,73 ± 16,82	320,17 ± 10,58	323,11 ± 39,00	352,78 ± 18,14	355,74 ± 8,34	365,50 ± 12,40
Mito-SOD	37,85 ± 3,37	41,50 ± 8,68	31,66 ± 2,21	37,67 ± 1,66	66,24 ± 3,47	63,59 ± 5,03
Cyto-APX	350,19 ± 8,04	350,85 ± 59,28	264,90 ± 5,33	368,44 ± 77,83*	308,89 ± 18,69	368,61 ± 58,69
Cyto-MDHAR	175,30 ± 30,65	115,72 ± 19,46	125,48 ± 20,43	123,30 ± 7,21	110,58 ± 13,80	131,05 ± 9,61
Cyto.Peroxi-DHAR	337,59 ± 57,82	222,45 ± 33,94*	236,81 ± 3,37	209,60 ± 27,90	320,25 ± 29,73	331,97 ± 51,19
Cyto.Peroxi-GR	54,07 ± 3,81	45,31 ± 3,36	42,74 ± 3,60	45,87 ± 4,79	47,28 ± 3,17	51,44 ± 4,35
Peroxi-APX	202,93 ± 6,77	228,89 ± 14,93	186,03 ± 37,71	194,93 ± 36,58	146,96 ± 10,00	147,12 ± 16,22
Peroxi-MDHAR	321,23 ± 27,36	210,55 ± 32,36*	214,14 ± 38,12	219,00 ± 34,55	187,40 ± 6,21	210,57 ± 15,20
Plastid-APX	69,39 ± 10,77	78,55 ± 12,53	86,90 ± 18,45	79,93 ± 9,43	78,98 ± 3,45	78,50 ± 13,95
Plastid-MDHAR	183,52 ± 3,48	126,70 ± 11,75	119,31 ± 24,13	119,39 ± 31,67	113,16 ± 13,34	108,77 ± 8,92
Mito-APX	12,91 ± 1,09	12,74 ± 1,62	11,04 ± 0,74	10,97 ± 0,67	17,58 ± 1,23	15,03 ± 0,45
Plasti.Mito APX	463,85 ± 64,87	485,99 ± 62,93	412,97 ± 33,90	320,70 ± 42,71*	485,60 ± 19,85	479,38 ± 31,96
Plastid.Mito-MDHAR	37,59 ± 1,41	31,87 ± 2,57	30,64 ± 3,58	23,70 ± 3,96	36,34 ± 2,33	29,24 ± 2,79
Plastid.Mito-DHAR	74,72 ± 1,90	64,10 ± 2,19	58,00 ± 6,74	58,51 ± 2,65	84,00 ± 4,49	76,51 ± 7,08
Plastid.Mito-GR	20,61 ± 0,53	18,70 ± 0,42	19,49 ± 3,00	16,26 ± 2,09	27,61 ± 3,16	21,67 ± 1,25
Cyto-GPX	281,79 ± 12,25	215,95 ± 19,61	167,52 ± 11,32	166,11 ± 24,33	150,36 ± 8,10	142,99 ± 19,07
Plastid-GPX	600,92 ± 70,57	596,20 ± 70,62	456,30 ± 64,64	608,16 ± 35,36*	354,34 ± 41,65	410,02 ± 40,90
Mito.Plastid-GPX	71,30 ± 13,27	61,50 ± 10,90	53,05 ± 11,50	60,15 ± 9,88	66,16 ± 11,30	59,52 ± 6,78
Cyto-Cat	53,93 ± 11,68	121,57 ± 11,92*	137,27 ± 19,46	103,75 ± 30,21	276,41 ± 6,68	301,11 ± 50,49
Peroxi-Cat	221,88 ± 18,51	236,83 ± 41,58	268,31 ± 18,89	239,12 ± 17,96	194,43 ± 13,88	298,63 ± 34,49*

Genotype: ir29				
	10 h		24 h	
	Control	Salt	Control	Salt
Cyto-SOD	472,05 ± 54,21	514,19 ± 40,33	653,64 ± 31,31	831,44 ± 92,09*
Peroxi-SOD	65,94 ± 8,43	67,43 ± 4,43	98,04 ± 4,18	88,33 ± 6,21
Plastid-SOD	500,30 ± 18,98	496,00 ± 24,94	264,18 ± 11,02	279,72 ± 7,94
Mito-SOD	95,00 ± 3,47	101,49 ± 10,47	37,87 ± 4,21	59,31 ± 11,24
Cyto-APX	457,85 ± 67,12	650,80 ± 79,01*	519,58 ± 25,31	582,76 ± 55,29
Cyto-MDHAR	136,65 ± 16,56	172,73 ± 25,49	173,30 ± 3,87	207,87 ± 32,49
Cyto.Peroxi-DHAR	370,82 ± 47,56	363,27 ± 16,48	220,34 ± 6,11	261,40 ± 29,08
Cyto.Peroxi-GR	57,22 ± 8,11	80,85 ± 21,77	43,19 ± 3,57	73,64 ± 18,19
Peroxi-APX	210,33 ± 14,71	204,01 ± 16,86	221,99 ± 25,01	206,24 ± 68,71
Peroxi-MDHAR	198,86 ± 20,41	228,23 ± 14,96	255,64 ± 5,80	289,95 ± 28,95
Plastid-APX	111,23 ± 20,91	93,58 ± 23,21	61,00 ± 4,28	52,18 ± 13,96
Plastid-MDHAR	100,75 ± 9,12	86,26 ± 15,99	111,35 ± 11,89	103,98 ± 2,76
Mito-APX	21,86 ± 0,28	16,90 ± 1,50	11,42 ± 0,97	12,05 ± 3,09
Plasti.Mito APX	579,91 ± 63,92	581,64 ± 72,34	400,38 ± 33,29	399,12 ± 44,57
Plastid.Mito-MDHAR	38,55 ± 5,29	30,77 ± 4,54	29,01 ± 2,96	21,91 ± 4,27
Plastid.Mito-DHAR	108,33 ± 6,28	92,47 ± 19,66	48,00 ± 2,46	41,63 ± 0,78
Plastid.Mito-GR	36,43 ± 2,46	30,84 ± 3,42	14,41 ± 1,41	16,15 ± 3,26
Cyto-GPX	49,67 ± 9,10	57,57 ± 11,77	213,44 ± 24,86	175,71 ± 22,04
Plastid-GPX	341,76 ± 98,27	542,14 ± 164,93*	931,33 ± 57,41	1146,16 ± 140,37*
Mito.Plastid-GPX	76,42 ± 18,53	89,83 ± 19,88	57,12 ± 6,95	71,94 ± 13,30
Cyto-Cat	283,92 ± 6,62	237,89 ± 32,72	38,29 ± 0,93	45,96 ± 3,72
Peroxi-Cat	134,98 ± 2,91	127,89 ± 45,78	149,71 ± 12,21	221,71 ± 41,19*
